# Artificial light at night does not alter heart rate or locomotor behaviour in Caribbean spiny lobster (*Panulirus argus*): insights into light pollution and physiological disturbance using biologgers

**DOI:** 10.1093/conphys/coaa097

**Published:** 2020-12-07

**Authors:** S Clay Steell, Steven J Cooke, Erika J Eliason

**Affiliations:** 1 Fish Ecology and Conservation Physiology Laboratory, Department of Biology, Carleton University, 1125 Colonel By Dr., Ottawa, ON K1S 5B6, Canada; 2 Department of Ecology, Evolution and Marine Biology, University of California, Santa Barbara, CA 93106, USA

**Keywords:** heart rate, accelerometer, biologging, spiny lobster, light pollution, stress

## Abstract

Light pollution is a rapidly growing threat to biodiversity, with many unknown or poorly understood effects on species and ecosystems spanning terrestrial and aquatic environments. Relative to other taxa, the effects of artificial light at night on aquatic invertebrates are poorly understood, despite the abundance and integral significance of invertebrates to marine and freshwater ecosystems. We affixed heart rate and acceleration biologgers to adult Caribbean spiny lobster (*Panulirus argus*), an ecologically, culturally and economically significant species in the western Atlantic ocean, to test the effect of artificial light at night on this species’ physiology and behaviour relative to appropriate controls. The experiment was conducted in a simulated reef mesocosm in The Bahamas with incandescent lighting used to illuminate it at 1 lux, approximating light levels offshore of urban areas. In the conditions tested here, artificial light at night was found to have no effect on heart rate or locomotor activity in *P. argus*. We observed a dissociation between activity and heart rate at both short-term and long-term temporal scales. Lobsters were more active at night and nocturnal activity was higher in trials closer to new moon; however, heart rate did not vary with diel or lunar cycle. There was less than 8% difference between daytime and night time average heart rate despite the average percentage of time spent active almost tripling in nights versus days, to 19.5% from 7.2%, respectively. Our findings suggest *P. argus* may have some resilience to low levels of light pollution, which warrants further research on aspects of this species’ life history, performance and fitness in the face of this potential anthropogenic disturbance.

## Teaser

Light pollution can disrupt animal health and behaviour; however, its effects on aquatic invertebrates are poorly understood. We found that unnatural light at night had no effect on heart rate or activity in Caribbean spiny lobster, suggesting this species and its fisheries may be somewhat resilient to light pollution.

## Introduction

Light pollution is a rapidly growing threat to terrestrial and aquatic biodiversity worldwide (Gaston *et al.*, 2013; Koen *et al.*, 2018). A growing body of research has elaborated the ways in which unnatural light at night impacts organisms and ecosystems, which include disruptions of sensory perception, diel cycle and ecological interactions (Hölker *et al.*, 2010; Davies *et al.*, 2016). Such disruptions are underpinned by physiological processes, the study of which can inform how organisms respond to light pollution, particularly when its effects are not visible to the naked eye (Navara and Nelson, 2007; Gaston *et al.*, 2013). Most research on the subject has focused on terrestrial systems; however, light pollution threatens marine and freshwater organisms and is less well understood in these aquatic environments (Davies *et al.*, 2016). Davies *et al.* (2014) estimated that 22% of coastal areas on Earth (excluding Antarctica) experience some degree of light pollution, which Kyba *et al.* (2017b) estimated is growing by 2.2% in area and brightness annually as coastal areas worldwide continue to rapidly urbanize (Neumann *et al.*, 2015).

Diverse biomarkers are used to assess how animals respond to extrinsic stimuli, including those of anthropogenic origin such as light pollution, which collectively contribute to allostatic load (McEwen and Wingfield, 2010). In the context of wild organisms, there is particular interest in understanding how anthropogenic disturbances influence performance, fitness and population-level ecological processes (Wikelski and Cooke, 2006, Dantzer et al., 2014). Light pollution has been found to disrupt hormone cycles or induce stress in several species of fish (inferred largely from glucocorticoids), such as European perch (*Perca fluviatilis*) (Brüning *et al.*, 2015, Brüning *et al.*, 2018), bonefish (*Albula vulpes*) (Szekeres *et al.*, 2017), Atlantic salmon (*Salmo salar*) (Migaud *et al.*, 2007) and baunco (*Girella laevifrons*) (Pulgar *et al.*, 2019), along with altering aquatic animal behaviour (Gaston *et al.*, 2015; Thums *et al.*, 2016; Fischer *et al.*, 2020) and ecosystem structure (Perkin *et al.*, 2011; Underwood *et al.*, 2017). Few studies have investigated the effect of light pollution on aquatic invertebrates, despite their abundance and integral significance in aquatic ecosystems. Moderate levels of artificial light at night (i.e. 4–15 lux) have been found to suppress activity and alter behaviour in four species of North American crayfish (Jackson and Moore, 2019; Fischer *et al.*, 2020), and the Japanese spiny lobster (*Panulirus japonicus*) has been reported to suppress activity at extremely low levels of artificial light at night (1.8 × 10^−4^ lux) (Nagata and Koike, 1997). We are aware of only one study to date that has measured hormones in an aquatic invertebrate subjected to artificial light at night in an attempt to understand if it contributed to allostatic load (Jackson and Moore, 2019), which found that it did not affect levels of haemolymph serotonin in two species of North American crayfish, despite disrupting their behaviour and activity levels. While hormone levels can reflect an animal’s status over a given period of time prior to sampling, other endpoints can be used to assess stress responses more continuously and at higher resolution, such as heart rate (*f*_H_) and locomotor activity. Moreover, so-called ‘stress hormones’ (i.e. glucocorticoids) are increasingly recognized as having diverse roles in organism function (MacDougall-Shackleton *et al.*, 2019).

Heart rate has been used as a measure of physiological status and allostatic load in a range of animal taxa, including the decapod crustaceans. Decapods have the most complex circulatory system among crustaceans, similar to that of basal vertebrates, and changes in *f*_H_ have been found to be a reliable indicator of physiological state in numerous decapod species (McGaw and Reiber, 2015). As in fish and other vertebrates, *f*_H_ often increases with exposure to stress; however, decapods may also undergo bradycardia (slowing of *f*_H_) or acardia (temporary cessation of *f*_H_) when exposed to a physiological challenge depending on its rapidity, duration and severity (Brown *et al.*, 2004; McLean and Todgham, 2015). The measurement of *f*_H_ in decapods has historically been confined to tethered or constrained animals; however, McGaw *et al.* (2018) developed a novel methodology for measuring *f*_H_ in free-moving decapods using *f*_H_ biologgers. In addition, advances in acceleration biologger technology (i.e. accelerometers) have made it possible to remotely measure activity in small, free-moving animals (Brown *et al.*, 2013; Cooke *et al.*, 2016), activity being a primary determinant of *f*_H_ in decapods as well as a measure of behavioural response to a stimulus (Bertelsen, 2013; Goldstein *et al.*, 2015; Gutzler *et al.*, 2015; Jury *et al.*, 2018).

In this study, we used heart rate biologgers along with tri-axial accelerometers to explore the physiological and behavioural response of Caribbean spiny lobster (*Panulirus argus*) to light pollution. *Panulirus argus* forms one of the most culturally and economically significant fisheries in the western Atlantic Ocean, employing over 60 000 people and generating more than $1 billion in revenue annually (Winterbottom *et al.*, 2012; FAO, 2018; CLME+, 2019). In The Bahamas, where *P. argus* is known as crawfish, the fishery is the largest and most valuable in the country (Smith and Zeller, 2013; Sherman *et al.*, 2018) and the Bahamian fishery is the largest in the species’ range (CLME+, 2019). While the Bahamian fishery was certified by the Marine Stewardship Council in 2018 (Gascoigne *et al.*, 2018), *P. argus* has had a ‘data deficient’ IUCN Red List status over its entire range since its last assessment in 2009 (Butler *et al.*, 2013) and its fisheries are likely overfished in many places (CRFM, 2011; Winterbottom *et al.*, 2012; WECAFC, 2018; CLME+, 2019). *Panulirus argus* is also an ecologically significant species, influencing community structure through predation, underpinning trophic linkages from chemosynthetic primary production and forming an important part of its predators’ diets (Smith and Herrkind, 1992; Higgs *et al.*, 2016; Gnanalingam and Butler IV, 2018). As a nocturnal generalist with a light-mediated circadian cycle of activity that forages in shallow nearshore areas, *P. argus* may be increasingly subjected to light pollution as coastal and offshore development near their habitat continues, and its heavily-exploited status may make this species particularly sensitive to emerging anthropogenic disturbances. To our knowledge, the effect of light pollution on any aspect of this species’ biology or ecology has not been investigated to date. As it has the potential to disrupt diel endocrine cycles that mediate physiological processes (Castanon-Cervantes *et al.*, 1999; McGaw and Reiber, 2015; Jackson and Moore, 2019), we hypothesized that artificial light at night would (1) increase *f*_H_ in *P. argus*, indicating that it is contributing to allostatic load, and (2) decrease its nocturnal activity as has been found in *P. japonicus* and other decapods, indicating a behavioural alteration (Nagata and Koike, 1997; Jackson and Moore, 2019; Fischer *et al.*, 2020).

## Methods

### Animal collection and husbandry

Lobsters (*n* = 36, mean body mass 499.7 ± 97 g, all data are reported as means ± SEM) were collected with SCUBA from patch reefs and forereefs between 3 and 15 m deep near Cape Eleuthera, The Bahamas (24°50′31 N, 76°20′40 W) between 31 December 2017 and 3 February 2018, using a curved pole to coax them out of reef crevices and into a hand net. Lobsters were held in outdoor circular 750-L tanks that were aerated and continuously supplied with fresh seawater (5 L min^−1^) at ambient temperature (mean = 23.4 ± 0.24°C) at the Cape Eleuthera Institute, which was less than 5 km from capture sites. Lobsters were held in laboratory conditions for a minimum of 3 days and held for a maximum of 14 days before experimentation. Lobsters were fed a combination of squid, mackerel and tilapia *ad libitum* during holding and were fasted for 48 hours before experimentation to ensure they were in a post-absorptive state and that *f*_H_ measurements were not confounded by elevated postprandial metabolism. While the duration of elevated postprandial metabolism is not known for *P. argus*, it can remain elevated for several days in other decapods at similar temperatures (Whiteley *et al.*, 2001; Perera *et al.*, 2007; McGaw and Reiber, 2015), and the duration of this experiment was shorter than the typical onset of starvation-associated declines in heart rate and metabolism in decapods (Ansell, 1973; Depledge, 1985). With the exception of one lobster that moulted during the third light treatment trial of this experiment (see below), all lobsters were in an intermoult period.

### Surgical and instrumentation protocol

**Figure 1:**
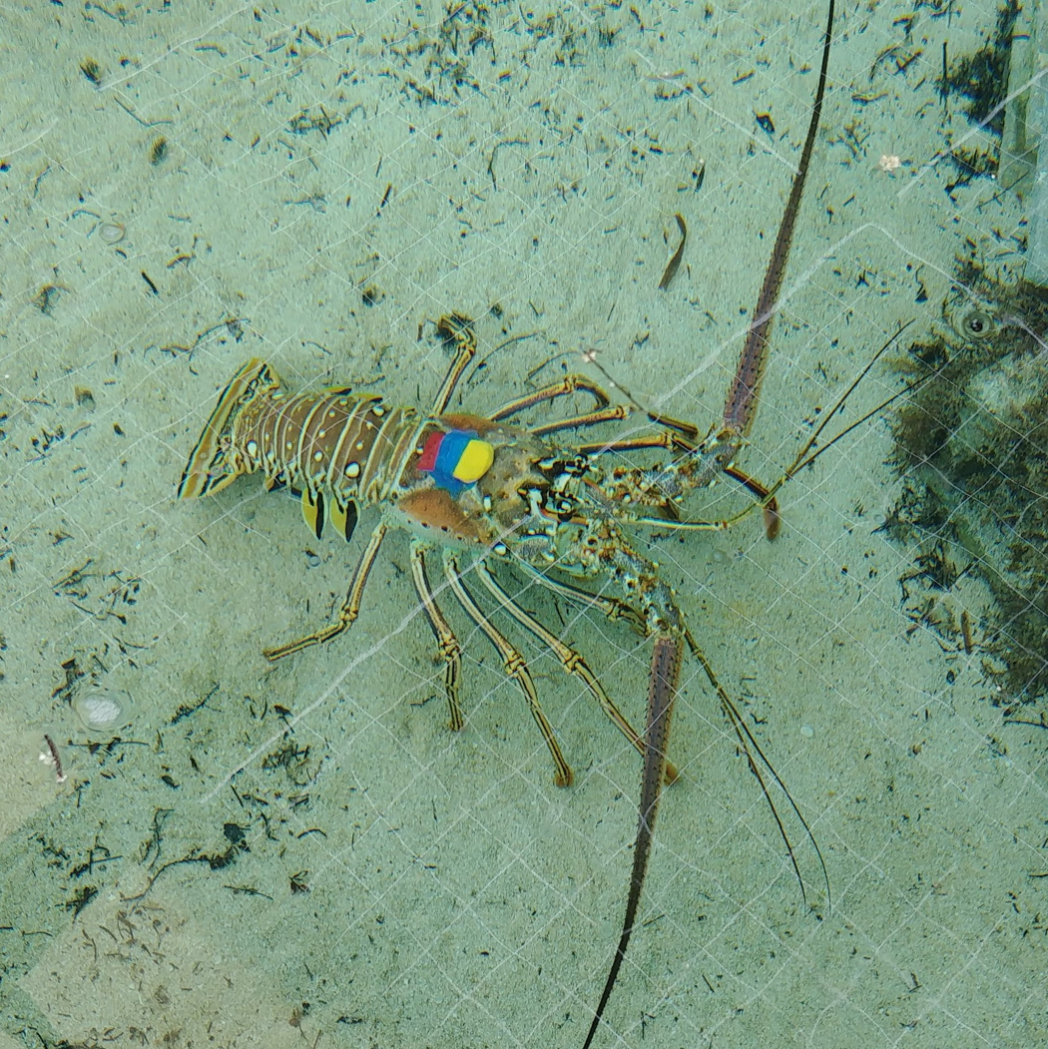
A lobster instrumented with a tri-axial accelerometer, affixed above a sheet of periphery wax covering the heart rate biologger.

The method used for affixing heart rate biologgers in this study was adapted from that in McGaw *et al.* (2018) (see online supplementary materials for a video). Briefly, lobsters were held by an assistant in a shallow tray of aerated seawater so that their gills were irrigated during the procedure, which took 11 minutes to complete on average. The best position to hold them was found to be with one hand arched over the head and the other hand firmly holding the tail so that it stayed curved, in which the lobster was unable to flex its tail and attempt escape. An oval-shaped section of the carapace was removed by shaving the edges of the area directly above the heart, which is clearly delimited in decapod crustaceans (McGaw *et al.*, 2018), with a handheld rotary tool (Dremel® 7300-N/8 MiniMite, Mt. Prospect, USA) fitted with an abrading burr attachment. This carapace section was then carefully removed with forceps, exposing a thin layer of dermis above the heart. Surgident® periphery wax (Heraues Kulzer, South Bend, USA) was formed into a ring and scored along its bottom edge with a teasing needle tool before applying cyanoacrylate glue and affixing it to the carapace surrounding the exposed section. Leadless heart rate and temperature loggers (DST micro-HRT, Star Oddi, Gardabaer, Iceland; 25.4 × 8.3 mm; 3.3 g in air) were placed on the dermis above the heart, with the two measurement electrodes in full contact with the tissue and the end with the grounding electrode facing posteriorly. The ring of periphery wax was then sculpted to hold the logger in place, and a flattened sheet of periphery wax was placed over it and merged with the ring to fully cover the logger and exposed dermis. A tri-axial accelerometer (Human Activity Monitor model X-16, Gulf Coast Data Concepts, Waveland, MS; 55.9 × 39.4 × 15.2 mm; 20 g in air) sealed with Plasti Dip® (Plasti Dip Intl, Blaine, USA) was secured above this flattened sheet with cyanoacrylate glue and a band of periphery wax (Fig. 1).

### Experimental setup and protocol

This study took place in a simulated shallow reef mesocosm at the Cape Eleuthera Institute. The mesocosm was constructed with a 3.7-m-diameter, 1.8-m-deep circular tank furnished with a sand bottom and cinderblock shelters collected from a nearshore rubble dump, as these environmental simulacra have been found to minimize the confounding effects of laboratory conditions on *f*_H_ measurement in other decapod crustacean species (Florey and Kriebel, 1974; Aagaard *et al.*, 1995; McGaw and Nancollas, 2018; McGaw *et al.*, 2018), and this species is commonly found in shallow patch reefs of a similar area and depth at high densities (Bertelsen 2013, Gutzler et al. 2015). The mesocosm was aerated and supplied with fresh seawater at approximately 1 l min^−1^, with the water drained and refilled between trials.

Heart rate loggers and tri-axial accelerometers were used to measure lobsters’ *f*_H_ and activity, respectively. Accelerometers were used in order to measure activity in total darkness, as opposed to other means of measuring activity such as video, as activity is one of the primary determinants of *f*_H_ in decapod crustaceans (McGaw and Reiber, 2015). Heart rate loggers were set to record *f*_H_ over a 5 second period (125 Hz) every 10 minutes and save electrocardiogram (ECG) data once an hour for validation purposes, with start time delayed by either 24 or 48 hours depending on measurement trial (see below) to save memory and battery. Accelerometers were set at 25 Hz and activated immediately following instrumentation.

**Figure 2:**
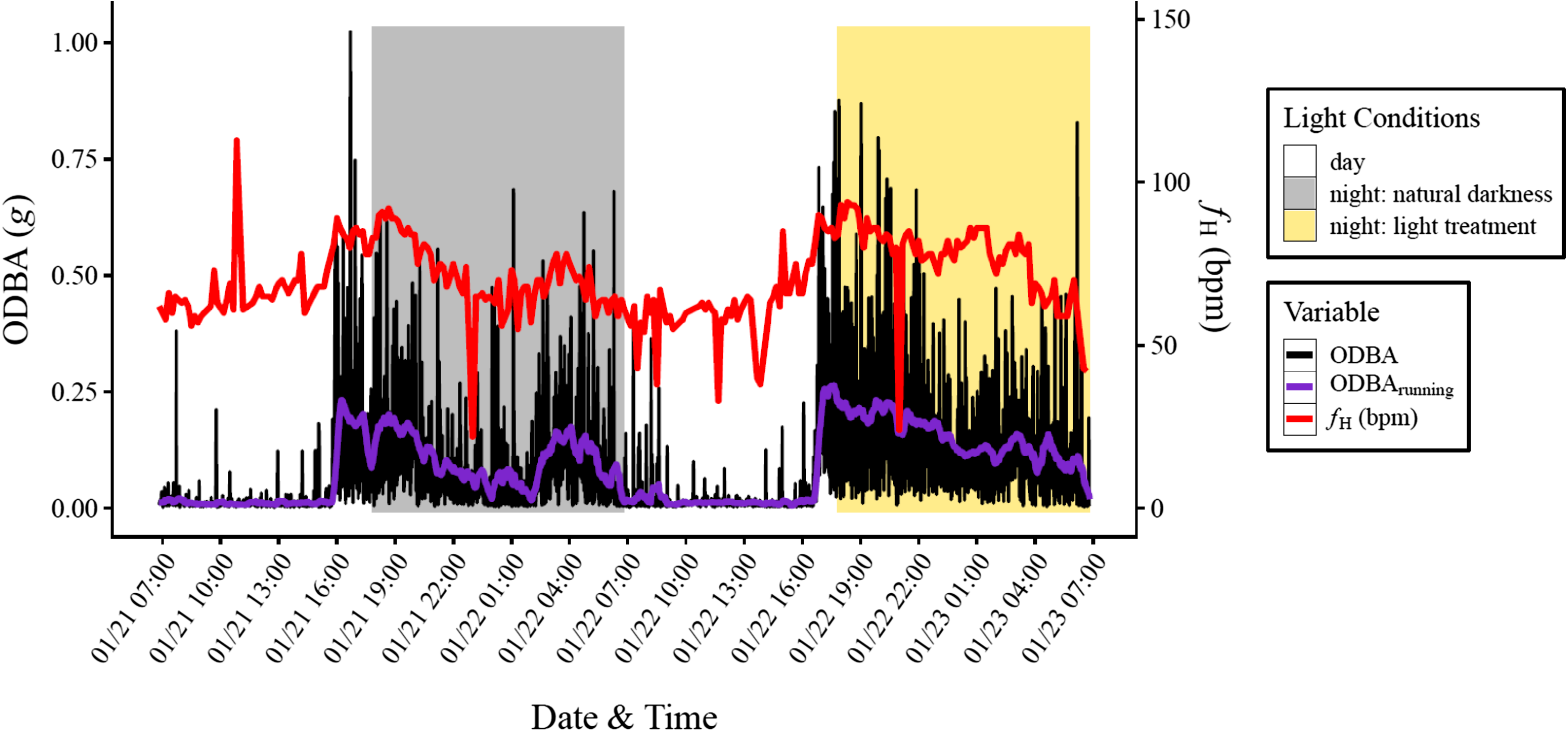
Examples of heart rate (*f*_H_), activity (overall dynamic body acceleration, or ODBA) and a running mean of activity (ODBA_running_) recorded in a lobster in Trial 2 (Jan 2018). Day and night were distinguished with sunrise and sunset times at 6:50 and 17:50, respectively, with the fourth night following instrumentation indirectly lit by an incandescent bulb for an illuminance of 1 lux at the mesocosm surface. ODBA was derived by summing the absolute difference between dynamic acceleration and static acceleration in each of the three axes recorded by the accelerometers, with static acceleration derived from a 6 second running mean. ODBA_running_ was derived as a 25-minute running mean of ODBA.

The experiment was carried out over 4 successive trials, each lasting 4 days and 4 nights. Each trial started immediately following instrumentation, with 8–10 lobsters placed in the mesocosm. A Surgery Recovery Trial was first conducted (*n* = 8) from 13 January to 17 January 2018 to ensure *f*_H_ and activity had stabilized by the third day following surgery and instrumentation, when we intended to start using the data in analysis. In this trial, the heart rate loggers’ start time was delayed by 24 hours. Three successive light treatment trials were then conducted, with Trial 1 from 20 January to 24 January 2018 (*n* = 9), Trial 2 from 24 January to 28 January 2018 (*n* = 10) and Trial 3 from 6 February to 10 February 2018 (*n* = 9) in which the fourth night was unnaturally lit to compare it to the third night, which was naturally dark (Fig. 2). The heart rate loggers’ start time was delayed by 48 hours in these three trials, and the fourth night was indirectly illuminated with a 60-watt incandescent light bulb positioned so that the illuminance at the surface of the mesocosm was 1 lux, measured with a handheld lux meter (Dr Meter®, Hisgadget Inc., Union City, CA, USA), turned on at 17:20, half an hour before sunset. An incandescent bulb was chosen as it approximates the spectra of high-pressure sodium lamps, the most common street lighting worldwide, and 1 lux was chosen as it approximates the ‘sky-glow’ found near urbanized coastal areas (Perkin *et al.*, 2014; Brüning *et al.*, 2015; Dąbrowski *et al.*, 2015). Natural night time illuminance was at or below the 0.1 lux lower detection limit of the lux meter, consistent with ground and satellite observations of light pollution and moonlight illuminance for the area (Falchi *et al.*, 2016; Kyba *et al.*, 2017a). Lunar cycle ranged between third quarter and new moon during the Surgery Recovery Trial, ranged between first quarter and full moon during Trials 1 and 2 and ranged between full moon and third quarter during Trial 3. Average temperatures for the Surgery Recovery Trial, Trial 1, Trial 2 and Trial 3 were 23.1 ± 0.1°C, 23.6 ± 0.1°C, 21.9 ± 0.1°C and 25.3 ± 0.1°C, respectively, with the difference between minimum and maximum temperature less than 2.7°C in all trials.

Following experimentation, the accelerometers and heart rate loggers were removed and a new sheet of periphery wax was used to cover the exposed tissue. Lobsters were allowed to recover in the wet laboratory and fed *ad libitum* for 48 hours before being released on reefs near where they were caught. McGaw *et al.* (2018) observed moulting in animals held in tanks following this procedure, after which the excised area of the shell regrew normally, suggesting released lobsters could soon recover. Such a recovery of the excised shell area was observed in the lobster that moulted during Trial 3. All work was carried out under the Cape Eleuthera Institute’s research permit from The Bahamas Department of Marine Resources.

### Data preparation

DST micro-HRT heart rate loggers automatically calculate a Quality Index (QI) for each measurement ranging from 0 (best) to 3 (worst) based on the ability of its algorithm to estimate *f*_H_ from an ECG recording. The loggers and this algorithm were designed for use in small mammals, and these *f*_H_ estimates and corresponding QI have not been validated in decapod crustaceans. To do so, ECG recordings were analysed manually using the Pattern Finder software (v. 1.11.0, Star Oddi, Gardabaer, Iceland). Three ECG recordings were randomly selected from each QI in each lobster in the Surgery Recovery Trial and compared to the loggers’ corresponding *f*_H_ estimates. Only *f*_H_ estimates with QI = 0 were found to be accurate, with an average accuracy of 98.3%, as opposed to the average accuracies of 75.9%, 76.7% and 63% of estimates with QI = 1, QI = 2 and QI = 3, respectively. Heart rate was therefore filtered to only include measurements of QI = 0. This resulted in temporal data gaps in some lobsters, for which *f*_H_ was measured manually using Pattern Finder from the ECG recordings saved hourly. As such, temporal gaps in *f*_H_ data were never longer than 1 hour. Lobsters that had only one measurement per hour for more than 6 consecutive hours were excluded from analysis, resulting in the exclusion of 12 lobsters out of the 36 total.

Accelerometer data were converted into overall dynamic body acceleration (ODBA) in *g* units by summing the absolute difference between dynamic acceleration and static acceleration in each of its three axes, with static acceleration derived from a 6 second running mean per the methodology of Shepard *et al.* (2008). Behaviour in a given moment was categorized with a 1 second running mean of ODBA, based on 2.5 hours of video recorded of an instrumented lobster’s movements, from three categories with non-overlapping ODBA thresholds: sitting (ODBA, ≤ 0.05 *g*), walking (ODBA, >0.05 and ≤1.5 *g*) or bursting (ODBA, >1.5) (i.e. when lobsters rapidly swim backwards by flapping their tail).

### Statistical analysis

Heart rate was averaged by day and night (*f*_H-diel_) for analysis, with day and night distinguished by sunrise and sunset times at 6:50 and 17:50, respectively, which minimized discrepancies in sunrise and sunset times across the trials to less than 15 minutes. Daily or nightly average heart rate (*f*_H-diel_) was analysed in ANCOVA models separately between the Surgery Recovery Trial (*n* = 6) and the light treatment trials (*n* = 14). These sample sizes were due to the 12 lobsters excluded for temporal gaps in *f*_H-diel_ and another 6 lobsters excluded for accelerometer failures. In the model for the Surgery Recovery Trial, the effect of sequential days and nights (day 2, night 2, day 3, night 3, day 4 and night 4) was tested on *f*_H-diel_, with daily or nightly average ODBA and body mass as covariates. In the model for the light treatment trials, the effects of sequential days and nights (day 3, night 3 with natural darkness, day 4 and night 4 with the light treatment) and trial (Trial 1, Trial 2 and Trial 3) were tested on *f*_H-diel_, with daily or nightly average ODBA and body mass as covariates. Sex could not be analysed in these models as it was unevenly distributed between trials, but there was no consistent difference between the sexes across trials and only a 1.1% difference in *f*_H-diel_ between them overall. Daily or nightly average heart rate (*f*_H-diel_) was log-10 transformed in the Surgery Recovery Trial model to meet assumptions.

Activity was analysed in ANOVA models separately between the Surgery Recovery Trial and the light treatment trials, with the percentage of time lobsters spent walking averaged by day and night (A_diel_) for analysis. Lobsters were found not to engage in bursting behaviour outside of the first few hours following instrumentation, so A_diel_ represented the total proportion of time lobsters were active in the times examined. Body mass was not included as a covariate in these models as it did not meet the assumption of linearity with the dependent variable, and as above sex was not included as it was unevenly distributed between trials. In the model for the Surgery Recovery Trial, the effect of sequential days and nights was tested on A_diel_. In the model for the light treatment trials, the effect of sequential days and nights and the effect of trial were tested on A_diel_. As these models did not test *f*_H_, they could include lobsters that were excluded from the *f*_H_ models above, so the sample sizes for the Surgery Recovery Trial model and light treatment trial model were 8 and 22, respectively. A_diel_ was log-10 transformed in both models to meet assumptions.

**Figure 3:**
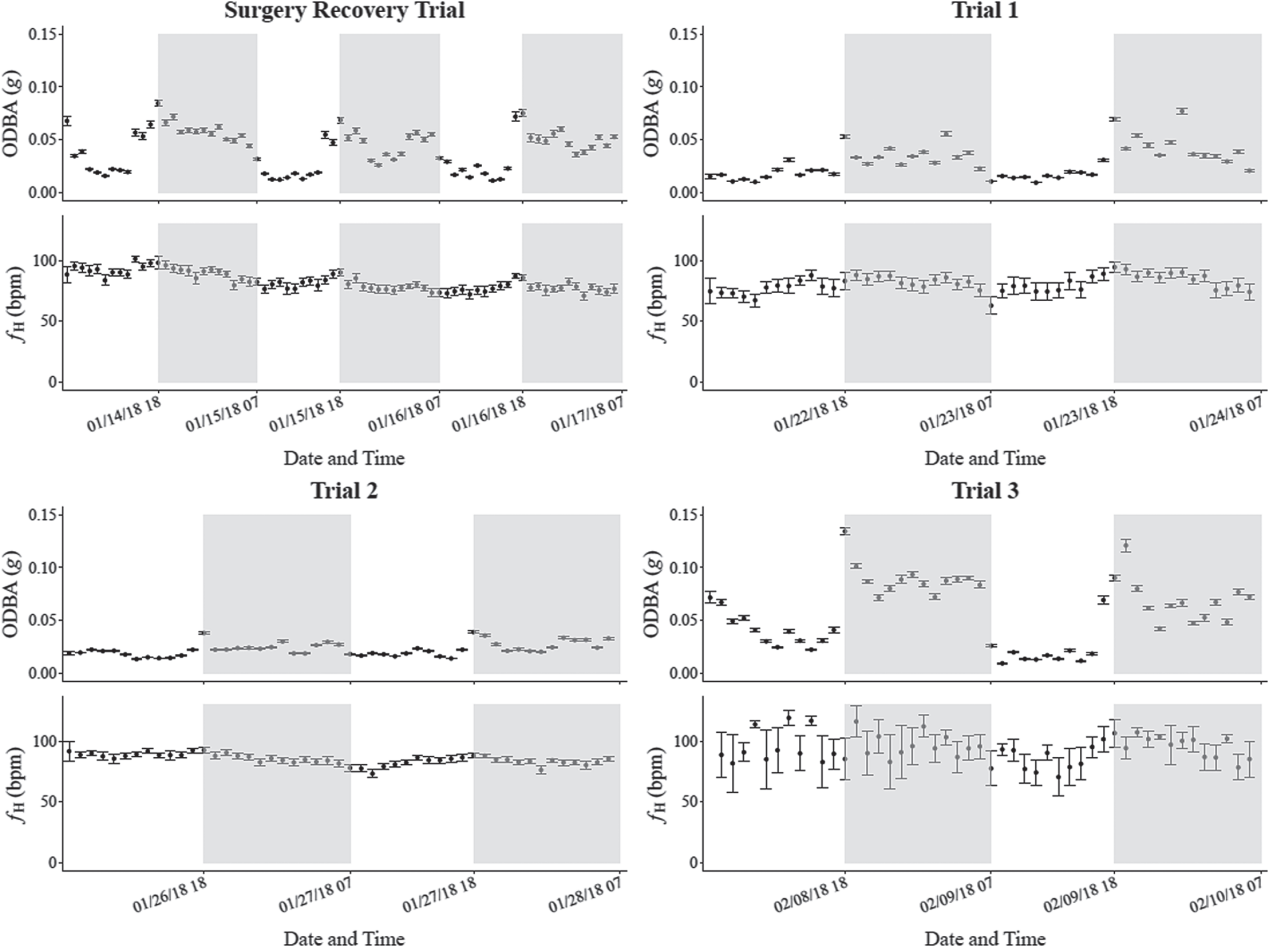
Heart rate (*f*_H_) and activity (overall dynamic body acceleration, or ODBA) by hour in each of the four trials in this study. ODBA was derived by summing the absolute difference between dynamic acceleration and static acceleration in each of the three axes recorded by the accelerometers, with static acceleration derived from a 6-second running mean. Shaded areas represent night time, with sunrise and sunset times at 6:50 and 17:50, respectively.

To assess whether *f*_H_ and activity over certain times of night were affected by the light treatment in ways that could be masked by averaging them in *f*_H-diel_ and A_diel_, respectively, *f*_H_ and ODBA were visualized by hourly increments in each trial (Fig. 3). Lobsters displayed a spike in activity around their nightly emergence, starting shortly before sunset and lasting 1–2 hours, in which ODBA was 15%–30% higher than activity over the rest of the night. The models above were therefore repeated on the average of *f*_H_ and the percentage of time lobsters spent walking between 17:20 and 19:20, denoted as *f*_H-dusk_ and A_dusk_, respectively, to compare *f*_H_ and activity during nightly emergence between night 3 dusk (natural darkness) and night 4 dusk (natural dusk in the Surgery Recovery Trial; unnaturally lit in the light treatment trials). 17:20 was chosen as the start of this temporal window as it was when the light was turned on half an hour before sunset in night 4 of the light treatment trials, with 19:20 chosen as its end because the spike in activity typically lasted 2 hours or less. In order to test the effect of activity on *f*_H_ at this 2-hour resolution, ODBA had to be averaged by a rolling mean leading up to a given *f*_H_ measurement, as *f*_H_ can remain elevated for several hours following bouts of activity in decapods (Rose *et al.*, 1998; Chabot and Webb, 2008; McGaw and Reiber, 2015). To determine the temporal relationship between activity and *f*_H_, a cross-correlation function was applied to the last 48 hours of ODBA and *f*_H_ measurements from individuals in the Surgery Recovery Trial, using the ‘ccf’ function in the R ‘stats’ package (version 3.4.4, R Foundation for Statistical Computing, Vienna, Austria). This cross-correlation function tests whether two time series correlate at temporal lags and whether these correlations are significant. The last 48 hours was chosen as the signal length as it was the only temporal window analysed where all lobsters had at least some significant correlations (e.g. compared to the last 24 hours or the third or fourth night following instrumentation, in which some lobsters had no significant correlations). It was found that, on average among significant correlations, *f*_H_ led ODBA at 25 ± 2.9 minutes, so a 25-minute running mean was applied to ODBA to use in the *f*_H-dusk_ and A_dusk_ models, henceforth referred to as ODBA_running_. *f*_H-dusk_ was analysed in ANCOVA models separately between the Surgery Recovery Trial (*n* = 6) and the light treatment trials (*n* = 14), with the effect of night 3 dusk versus night 4 dusk tested on *f*_H-diel_, the average of ODBA_running_ over dusk and body mass included as covariates, and trial included as a variable in the model for the light treatment trials. A_dusk_ was analysed in ANOVA models separately between the Surgery Recovery Trial (*n* = 8) and the light treatment trials (*n* = 22). The effect of night 3 dusk versus night 4 dusk was tested on A_dusk_, with trial included as a variable in the model for the light treatment trials. Body mass was not included as a covariate in the A_dusk_ models as it did not meet the assumption of linearity with the dependent variable. A_dusk_ was log-10 transformed in both models to meet assumptions.

Interactions were tested in all models by including interaction terms, and if an interaction occurred models were separated to analyse the difference between night 3 (natural darkness) and night 4 (light treatment) separately from the difference between day 3 and day 4. The effect of sequential days and nights and the effect of trial on both *f*_H-diel_ and A_diel_ interacted significantly (*P* = 0.04 for both), so separate ANCOVA models were run for each comparing night 3 to night 4 and day 3 to day 4. Post hoc Tukey HSD analyses were run for each model to determine which factor levels were driving significant differences. All statistical tests were performed in R (R version 3.4.4, RStudio Inc., Boston, MA, USA).

## Results

### Surgery recovery trial

**Figure 4:**
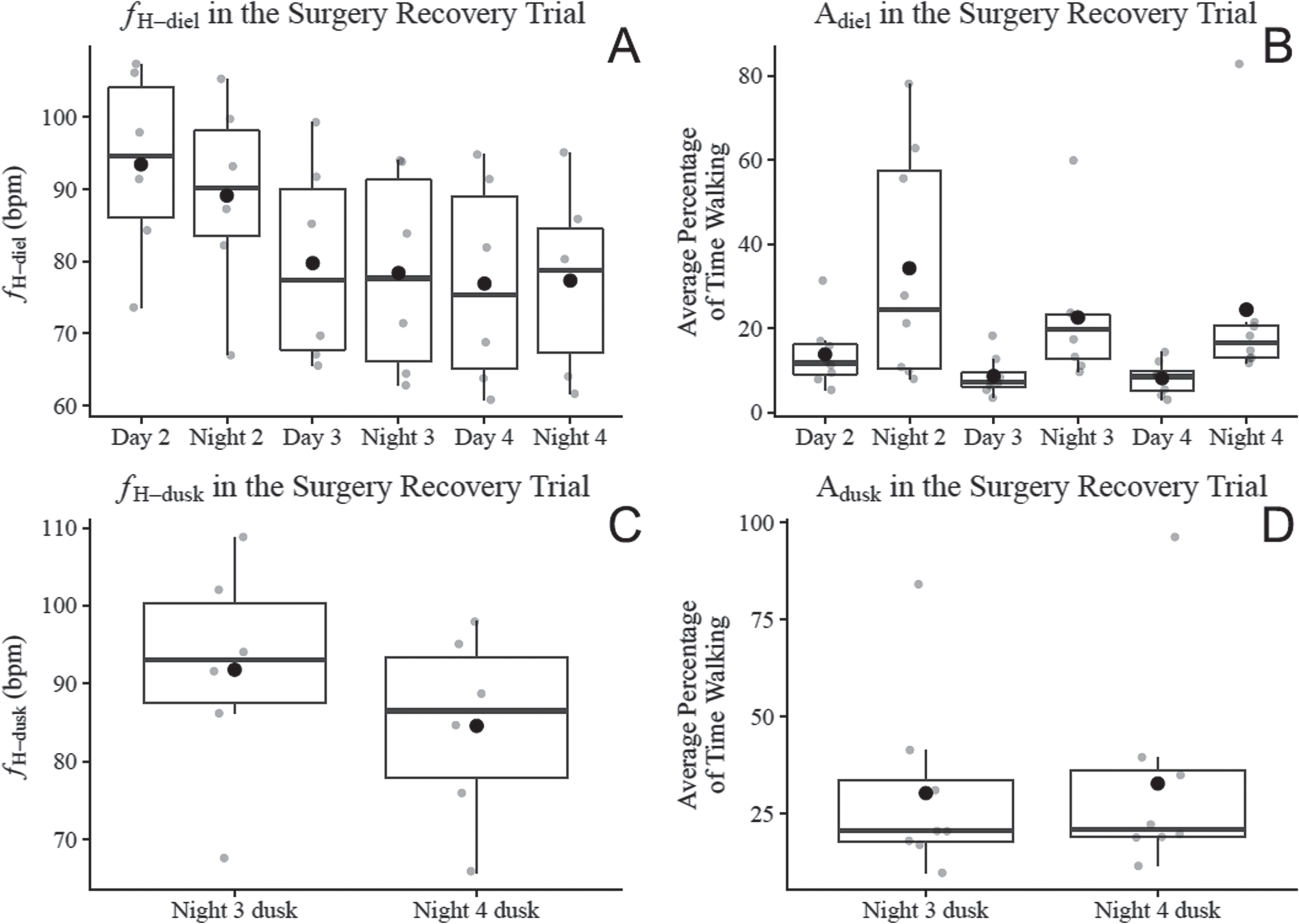
Daily and nightly average heart rate (*f*_H-diel_) and the percentage of time lobsters spent walking (A_diel_) over the surgery recover trial (A and B, respectively) and the average of *f*_H_ and the percentage of time lobsters spent walking between 17:20 and 19:20 (*f*_H-dusk_ and A_dusk_, respectively) between night 3 dusk and night 4 dusk in the Surgery Recovery Trial (C and D, respectively). Filled black points represent means, and horizontal black bars represent medians. Day and night were distinguished with sunrise and sunset times at 6:50 and 17:50, respectively, with the fourth night following instrumentation indirectly lit by an incandescent bulb for an illuminance of 1 lux at the mesocosm surface. The percentage of time lobsters spent walking was determined through the calibration of a 2.5-hour video of an accelerometer-instrumented lobster, which determined that ODBA recordings between 0.05 and 1.5 *g* corresponded to walking. ODBA was derived by summing the absolute difference between dynamic acceleration and static acceleration in each of the three axes recorded by the accelerometers, with static acceleration derived from a 6-second running mean.

In the Surgery Recovery Trial, *f*_H-diel_ and A_diel_ had stabilized by the third day following instrumentation (Fig. 4A, 4B). Post hoc testing found no significant difference in *f*_H-diel_ between day 3, night 3, day 4 and night 4. Daily or nightly average ODBA did not significantly affect *f*_H-diel_, and *f*_H-diel_ significantly declined with body mass (*P* < 0.001; see below for scaling exponents and coefficients). Mean *f*_H-diel_ between the third day and fourth night following instrumentation was 78.1 bpm in the surgery recovery trial.

The percentage of time lobsters spent walking (A_diel_) significantly differed over time (*P* < 0.001), however unlike *f*_H-diel_, post hoc testing found no significant difference in A_diel_ between day 2, day 3 or day 4, or between night 2, night 3 or night 4. According to post hoc testing, A_diel_ approached being significantly higher in night 3 than day 3 (*P* = 0.06) and was significantly higher in night 4 than day 4 (*P* = 0.04). On average, lobsters in the Surgery Recovery Trial spent 23.4% of the night walking, compared to 8.4% of the day walking (Fig. 4B).

Significant correlations between *f*_H_ and ODBA were relatively weak, between *r* = 0.12 and *r* = 0.14. According to the cross-correlation function run on *f*_H_ and ODBA in the last 48 hours of the Surgery Recovery Trial, *f*_H_ correlated with ODBA at a 25 ± 2.9 minute lag, on average among significant correlations; however, this lag ranged from 14 to 35 minutes across individuals.

The results from the models for *f*_H-dusk_ and A_dusk_ were consistent with those for *f*_H-diel_ and A_diel_. There was no significant difference in *f*_H-dusk_ or A_dusk_ between night 3 dusk and night 4 dusk (Fig. 4C, 4D). The average of ODBA_running_ over dusk had no effect on *f*_H-dusk_.

### Light treatment trials

**Figure 5:**
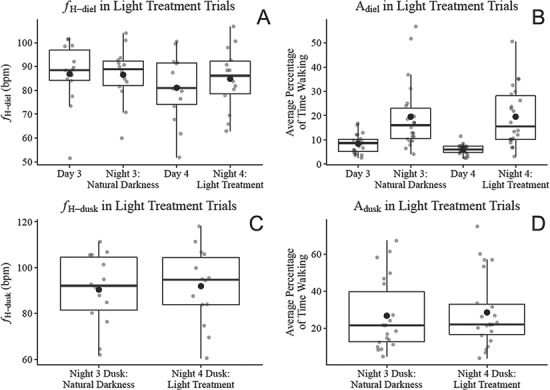
Daily and nightly average heart rate (*f*_H-diel_) and the percentage of time lobsters spent walking (A_diel_) over the light treatment trials (A and B, respectively) and the average of *f*_H_ and the percentage of time lobsters spent walking between 17:20 and 19:20 (*f*_H-dusk_ and A_dusk_, respectively) between night 3 dusk and night 4 dusk in the light treatment trials (C and D, respectively). Filled black points represent means, and horizontal black bars represent medians. Day and night were distinguished with sunrise and sunset times at 6:50 and 17:50, respectively, with the fourth night following instrumentation indirectly lit by an incandescent bulb for an illuminance of 1 lux at the mesocosm surface. The percentage of time lobsters spent walking was determined through the calibration of a 2.5-hour video of an accelerometer-instrumented lobster, which determined that ODBA recordings between 0.05 and 1.5 *g* corresponded to walking. ODBA was derived by summing the absolute difference between dynamic acceleration and static acceleration in each of the three axes recorded by the accelerometers, with static acceleration derived from a 6-second running mean.

**Figure 6:**
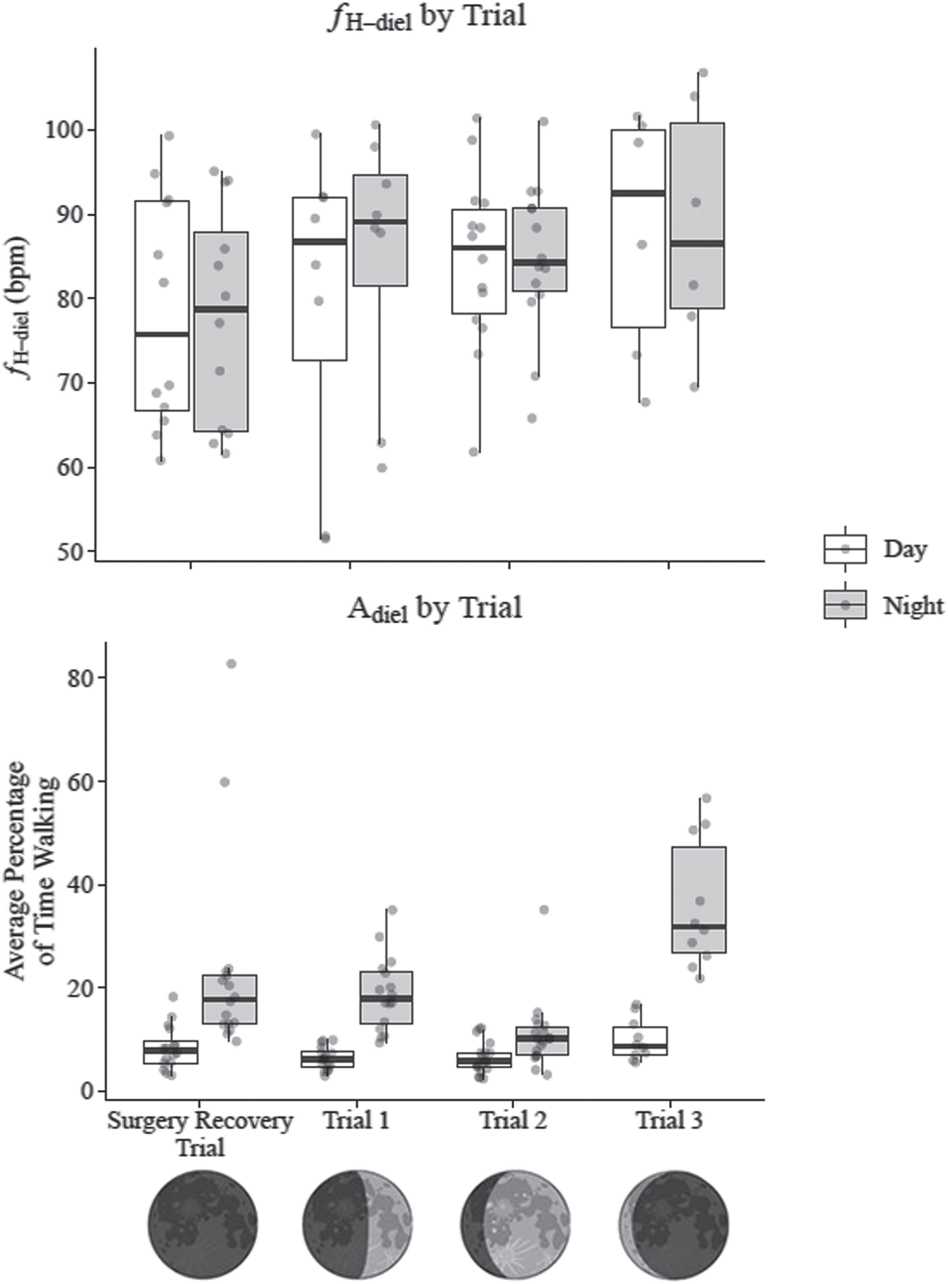
Daily and nightly average heart rate (*f*H_diel_) and the percentage of time lobsters spent walking (A_diel_) by trial, with an illustration of lunar cycle phase depicted below the x-axis. Dates for the Surgery Recovery Trial, Trial 1, Trial 2 and Trial 3 were 13–17 January 2018, 20–24 January 2018, 24–28 January 2018 and 6–10 February 2018, respectively. Lunar cycle phase on the final night of the Surgery Recovery Trial, Trial 1, Trial 2 and Trial 3 was new moon, first quarter, waxing gibbous (3 days before full moon) and waning crescent (3 days after third quarter). Day and night were distinguished with sunrise and sunset times at 6:50 and 17:50, respectively. Data plotted for the Surgery Recovery Trial include only that from day 3 to night 4 in order to be consistent with Trials 1 to 3. The percentage of time lobsters spent walking was determined through the calibration of a 2.5-hour video of an accelerometer-instrumented lobster, which determined that ODBA recordings between 0.05 and 1.5 *g* corresponded to walking. ODBA was derived by summing the absolute difference between dynamic acceleration and static acceleration in each of the three axes recorded by the accelerometers, with static acceleration derived from a 6-second running mean.

Daily or nightly average heart rate (*f*_H-diel_) did not significantly differ between night 4 (light treatment) and night 3 (natural darkness), and did not significantly differ between day 3 and day 4 (Fig. 5A). Over the course of the experiment, trial had no significant effect on *f*_H-diel_ in either model. Though not tested for significance due to model separation, there was less than an 8% difference in *f*_H-diel_ between nights and the preceding day in all trials, with no consistency in nights having higher or lower *f*_H-diel_ than days (Fig. 6). Daily or nightly average ODBA did not significantly affect *f*_H-diel_ in either model. Daily or nightly average heart rate (*f*_H-diel_) significantly declined with body mass (p = 0.002) in the model comparing nights and approached significantly declining in the model comparing days (p = 0.056). The scaling exponent (b) and coefficient (a) between log_e_-transformed body mass (kg) and log_e_-transformed *f*_H-diel_ (bpm) from day 3 to night 4 in all trials was b = −0.301 and a = 4.076, respectively. Mean *f*_H-diel_ over Trial 1, Trial 2 and Trial 3 was 82.6, 84.7 and 88.3 bpm, respectively.

The average daily or nightly percentage of time lobster spent walking (A_diel_) did not differ significantly between night 4 (light treatment) and night 3 (natural darkness) (Fig. 5B). Though not tested for significance due to model separation, lobsters were more active during nights than days, on average spending 19.5% of nights walking versus 7.2% of days walking. Over the course of the experiment, trial significantly affected activity in both days (*P* = 0.012) and nights (*P* < 0.001). In nights, according to post hoc testing, A_diel_ was significantly higher in Trial 3 than in Trial 1 (*P* = 0.002) or Trial 2 (*P* < 0.001) and was significantly lower in Trial 2 than Trial 1 (*P* < 0.001). In days, according to post hoc testing, A_diel_ was significantly higher in Trial 3 than Trial 1 (*P* = 0.03) or Trial 2 (*P* = 0.014), whereas Trial 1 and Trial 2 did not significantly differ. On average, lobsters walked 12.6% of the time in the Trial 1, 8.7% of the time in Trial 2 and 23% of the time in Trial 3 (Fig. 6).

Results from the models for *f*_H-dusk_ and A_dusk_ were consistent with those for *f*_H-diel_ and A_diel_. There was no significant difference in *f*_H-dusk_ or A_dusk_ between night 4 dusk (artificial light) and night 3 dusk (natural light) (Fig. 5C, 5B). The average of ODBA_running_ over dusk had no effect on *f*_H-dusk_. Over the course of the experiment, trial had no significant effect on *f*_H-dusk_, whereas A_dusk_ significantly differed between trials (p < 0.001) with the same pattern as A_diel_. According to post hoc testing, A_dusk_ was significantly higher in Trial 3 than in Trial 1 (p < 0.001) or Trial 2 (p < 0.001), and was significantly lower in Trial 2 than Trial 1 (p < 0.001). On average, lobsters spent 25.7% of dusk walking in Trial 1, 14.2% in Trial 2 and 55.3% in Trial 3.

#### Discussion

As unnatural light at night has been found to disrupt endocrine cycles and induce glucocorticoid-mediated stress responses in a suite of fish and terrestrial species (e.g. Gaston *et al.*, 2013; Brüning *et al.*, 2015; Szekeres *et al.*, 2017; Pulgar *et al.*, 2019), as well as alter behaviour in several species of decapod crustaceans (Nagata and Koike, 1997; Jackson and Moore, 2019; Fischer *et al.*, 2020), we hypothesized that *P. argus* would display a cardiac and behavioural response to its exposure. Contrary to our expectation, we found no change in *f*_H_ or activity between natural night time and night time artificially lit at 1 lux to approximate light pollution near urban areas, nor between nightly emergence around sunset with and without this light treatment. The *f*_H_ and diel levels of activity we observed were in line with past measurements in this species (Maynard, 1960; McGaw *et al.*, 2018) and those reported for wild *P. argus* affixed with accelerometers in the Florida Keys (Gutzler *et al.*, 2015), respectively, indicating that our mesocosm conditions or experimental protocol did not confound the lobsters’ regular physiology or phenology. To appropriately interpret these findings, we must consider the insights and limitations our observations provide into the relationships between *f*_H_, cardiac output, physiological disturbance and behaviour in this species.

We observed a potential dissociation between *f*_H_ and cardiac output, necessitating a nuanced consideration of how our *f*_H_ measurements may reflect a stress response to artificial light at night. In our models for *f*_H-diel_, we used daily or nightly average ODBA as a covariate to account for the effect of locomotor activity on *f*_H_, and in our models for *f*_H-dusk_, we used a 25-minute running mean of ODBA (ODBA_running_) as a covariate to account for the effect of recent locomotor activity on a narrower range of *f*_H_ readings. Contrary to precedence showing a positive relationship between *f*_H_ and activity in decapods due to the energetic demands imposed by locomotion (Rose *et al.*, 2001; Chabot and Webb, 2008; McGaw and Reiber, 2015), we observed a dissociation between these characteristics. Daily or nightly average ODBA did not significantly affect *f*_H-diel_, ODBA_running_ did not significantly affect *f*_H-dusk_, and even the best correlations between ODBA and *f*_H_ identified by a cross-correlation function and used to derive the 25-minute running mean for ODBA_running_ were relatively weak, between *r* = 0.12 and *r* = 0.14. As such, *f*_H-diel_ hardly differed between nights and days despite a marked increase in activity in nights versus days, nor between trials despite differences in nocturnal activity over lunar cycle (see below). It is possible that cardiac output does not increase following locomotor activity in *P. argus*, as such a dissociation has been reported in green crab (*Carcinus maenas*) and American lobster (*Homarus americanus*), in which *f*_H_ sometimes increased before the onset of locomotion (Aagaard *et al.*, 1995; O’Grady *et al.*, 2001). A more likely explanation for the dissociation we observed, however, is that cardiac stroke volume varied in response to locomotion rather than *f*_H_, as *f*_H_ and stroke volume can vary independently in decapods to determine overall cardiac output (McMahon and Burnett 1990, McGaw *et al.*, 1994; McGaw and McMahon, 1996). While *f*_H_ has been found to be the primary determinant of cardiac output during locomotion in European lobster (*Homarus vulgaris*), blue crab (*Callinectes sapidus*) and *C. maenas* (Booth *et al.*, 1982; Hamilton, 1987; Hamilton and Houlihan, 1992), stroke volume has been found to be a greater determinant of cardiac output than *f*_H_ during locomotion in Dungeness crab (*Cancer magister*) (McMahon *et al.*, 1979; Wachter and McMahon, 1996). The relative significance of *f*_H_ versus stroke volume to cardiac output may also change with locomotor activity, as found in *H. americanus*, where stroke volume becomes a greater determinant of cardiac output than *f*_H_ only at higher walking speeds (Rose *et al.*, 2001). The heart rate biologgers we used cannot measure stroke volume so we can only speculate, but the sustained periods of locomotion we observed with little to no change in *f*_H_ suggest *P. argus* actively modulates stroke volume to compensate for the energetic demands and required cardiac output of movement and activity.

In the absence of locomotion, changes in the balance of neurohormones that mediate cardiac output generally act on *f*_H_ more than stroke volume in decapods (McGaw *et al.*, 1995; McGaw and Reiber, 2015). Artificial light at night can disrupt the circadian cycles of other neurohormones, such as melatonin or serotonin, which can have cascading impacts on these cardiac-mediating neurohormones (Castanon-Cervantes *et al.*, 1999; McGaw and Reiber, 2015; Jackson and Moore, 2019). A dissociation between *f*_H_ and a glucocorticoid-mediated stress response in this species is therefore unlikely, despite the dissociation we observed between *f*_H_ and locomotion, as such a response would likely be reflected in *f*_H_ before stroke volume. As we observed no effect of an artificial light treatment on *f*_H_ or activity, we may assume that under the conditions tested here, artificial light at night does not represent a meaningful challenge to allostatic load or the circadian rhythm of *f*_H_ in *P. argus*. This is partly consistent with the findings of Jackson and Moore (2019), who found no effect of artificial light at night on haemolymph serotonin in two species of freshwater crayfish, rusty crayfish (*Faxonius rusticus*) and virile crayfish (*Faxonious virilis*). It is possible that our light treatment disrupted the lobsters’ endocrine balance at a magnitude small enough not to cause a change in *f*_H_ or behaviour; however, it is unlikely such a small change would have contributed meaningfully to allostatic load (Dantzer *et al.*, 2014; McEwen and Wingfield, 2010). Our findings that artificial light at night does not disrupt this species’ regular activity levels or nightly emergence differ from past studies reporting suppression of activity in other decapods species exposed to artificial light at night, such as *P. japonicus*, another shallow-water panulirid that was sensitive to illuminance as low as 1.8 × 10^−4^ lux (Nagata and Koike, 1997). This suggests that behavioural responses to light pollution may be species-specific in decapods, with *P. argus* displaying a lower sensitivity than *P. japonicus* or the four freshwater crayfish species analysed by Jackson and Moore (2019) and Fischer *et al.* (2020), however further research on these species using lower-intensity light levels found near urban areas would be necessary to test such a hypothesis.

Altogether, our findings suggest *P. argus* may be physiologically resilient to artificial light at night, and may be less impacted by light pollution at a physiological and behavioural level compared to previously-analysed fish and terrestrial species (Gaston *et al.*, 2013; Brüning *et al.*, 2015; Szekeres *et al.*, 2017; Pulgar *et al.*, 2019). This would mean that light pollution effects do not manifest in sublethal impacts to performance or fitness in *P. argus*, as has been found for other anthropogenic disturbances in this species like fishery interactions (Vermeer, 1987; Parsons and Eggleston, 2005; Maxwell *et al.*, 2009), and that its fisheries may not be as threatened by light pollution as those for other species. Uncertainties remain in how light pollution fully impacts *P. argus* biology and ecology, however, such as how their predators and prey are affected and how this could impact trophic interactions (Higgs *et al.*, 2016; Underwood *et al.*, 2017), whether ecosystem processes in *P. argus* habitat are affected in ways that manifest in performance or fitness impacts (Navara and Nelson, 2007; Boudreau and Worm, 2012; Ayalon *et al.*, 2019), how the strongly lunar-mediated planktonic larval distribution and settlement of *P. argus* are affected (Eggleston *et al.*, 1998), or whether long-term exposure to artificial light at night manifests in a more chronic physiological response. Answering these questions would be necessary to understand the vulnerability of *P. argus* and the sustainability of its fisheries in the face of light pollution, however our findings offer some preliminary insight into its resiliency to this anthropogenic threat. This may be particularly relevant as marine protected areas (MPAs) are increasingly utilized to conserve marine species and promote sustainable fisheries, including MPAs proposed to facilitate *P. argus* conservation (Lipcius *et al.*, 2001; CRFM, 2011; Bertelsen, 2013; WECAFC, 2018), as light pollution is increasingly encroaching on MPAs globally and may impact their efficacy (Davies *et al.*, 2016).

An incidental observation in this study was that nocturnal activity in *P. argus* varied over the lunar cycle, with a marked suppression in Trial 2 just prior to full moon. While the increase in activity from Trial 2 (close to full moon) to Trial 3 (close to new moon) may have been augmented by a 3.4°C increase in average temperature, the decline in activity from Trial 1 (first quarter) to Trial 2 was accompanied by a more subtle cooling of 1.7°C. Temperature changes at such small magnitudes typically do not influence movement in other decapod species, suggesting these changes in activity could be due to lunar cycle (MacDiarmid *et al.*, 1991; González-Gurriarán and Freire, 1994; Hines *et al.*, 1995; González-Gurriarán *et al.*, 2002). Fishers have anecdotally reported higher catches of *P. argus* around new moon, however the few studies conducted on the topic have produced conflicting results. Lopeztegui *et al.* (2011) found no correlation between lunar cycle and *P. argus* landings in Cuba, whereas Sutcliffe Jr (1956) reported higher landings during phases of low moonlight in Bermuda. Using acoustic telemetry, Bertelsen (2013) found that one-third of *P. argus* tagged on patch reefs in the Florida Keys were significantly more active during new moon and phases of low moonlight than during full moon, with a higher likelihood of entering fishers’ traps. Why such a relationship between nocturnal activity and lunar cycle occurs and why it may only manifest in some individuals or some places is unclear. Lunar cycle has been found to regulate moulting in other decapods, with ornate rock lobster (*Panulirus ornatus*), blue crab (*C. sapidus*) and European green crab (*C. maenas*) moulting during or close to full moons (Ryer *et al.*, 1990; Skewes *et al.*, 1994; Zeng *et al.*, 1999). Moult synchronization may lower the individual odds of predation, cannibalism, or interspecific competition for shelter during this vulnerable time, but we are only aware of evidence for *P. argus* synchronizing moults to photoperiod and temperature (Lipcius and Herrnkind, 1982, 1987; Quackenbush and Herrnkind, 1983), and not lunar cycle. While lower nocturnal activity would be expected during moult synchronization, we did not observe any moults during Trial 2 (the closest trial to full moon) or in lobsters held for acclimation and release before, during, and after full moon. As such, *P. argus* may regulate moulting independently of lunar cycle, as found in southern rock lobster (*Jasus edwardsii*) (MacDiarmid *et al.*, 1991), and other factors may explain the suppression of activity we observed near full moon. Light from the full moon may make *P. argus* more vulnerable to predation, as for the crab *Neohelice granulate* (López and Rodríguez, 1998), or tides closer to the full moon may create stronger currents that deter activity. Regardless of the reasons, lunar periodicity in capture likelihood, trap encounter, or other measures of fishing mortality are not currently incorporated into *P. argus* management in The Bahamas or in other countries (Butler *et al.*, 2013; Gascoigne *et al.*, 2018; WECAFC, 2018; CLME+, 2019). Further research on this topic is warranted to determine drivers of *P. argus* lunar periodicity in movement and activity and whether this information can be used to better manage the species and its fisheries (Srisurichan *et al.*, 2005; Lopeztegui *et al.*, 2011; Bertelsen, 2013).

In conclusion, we found evidence that artificial light at night does not alter heart rate or locomotor activity in *P. argus*, indicating that this species may be relatively resilient to light pollution. We used incandescent light at 1 lux to approximate the typical illuminance and spectra of light pollution in urbanized nearshore areas (Perkin *et al.*, 2014; Brüning *et al.*, 2015); however, further research on different light spectra and intensities would be valuable to determine the impact of increasingly-used LED street lighting and high-intensity point sources of offshore light pollution like oil platforms or ship anchorages (Migaud *et al.*, 2007; Aubrecht *et al.*, 2010; Fischer *et al.*, 2020). By pairing two biologging devices equipped with heart rate and acceleration sensors, we determined the prospects and limitations of this approach for studying the physiology and behaviour of wild or free-moving decapod crustaceans. As we were limited to measuring *f*_H_, further research is warranted to explore cardiac function in *P. argus*, and how *f*_H_ and stroke volume change in response to light pollution and other forms of anthropogenic disturbance. Such an approach would likely require using methods like pulsed Doppler, impedance conversion, or infrared transmission, however, which require varying degrees of animal constraint or tethering that can confound measurements of cardiac function (Aagaard *et al.*, 1995; McGaw and Nancollas, 2018; McGaw *et al.*, 2018). The heart rate biologgers we used therefore offer a compelling means of measuring *f*_H_ in free-moving or wild decapods, but may be limited in the insight they offer by their inability to measure stroke volume. Their applicability may vary with species, as some decapods exhibit a more predictable relationship between *f*_H_ and stroke volume than others (Hamilton, 1987; Hamilton and Houlihan, 1992; Rose *et al.*, 2001; McGaw and Reiber, 2015). Pre-existing information on this relationship exists for relatively few decapod species, meaning future use of these biologgers in other species must balance the limitations of using solely *f*_H_ measurements with the value of understanding their physiology in the face of anthropogenic disturbances. Another consideration is that these biologgers must be retrieved to download collected data, which may be challenging in highly mobile species like *P. argus*. While a similar study in the wild with *P. argus* would be valuable, our findings demonstrate that conditions in a mid-sized mesocosm did not confound this species’ regular phenology or *f*_H_ (Maynard, 1960; Gutzler *et al.*, 2015; McGaw et al., 2018). Despite these limitations, this technology shows promise for understanding the biology of decapod species threatened by anthropogenic disturbance in a scalable and relatively low-cost way (McGaw *et al.*, 2018).

## Funding

This work was supported by the Natural Sciences and Engineering Research Council of Canada and the Canada Research Chairs Program [to S.J.C.] and by the University of California, Santa Barbara [to E.J.E.].
